# Illicit Alcohol: Public Health Risk of Methanol Poisoning and Policy Mitigation Strategies

**DOI:** 10.3390/foods10071625

**Published:** 2021-07-13

**Authors:** Louise Manning, Aleksandra Kowalska

**Affiliations:** 1 School of Agriculture, Food and the Environment, Royal Agricultural University, Stroud Road, Cirencester GL7 6JS, UK; 2 Institute of Economics and Finance, Maria Curie-Skłodowska University, pl. Marii Curie-Skłodowskiej 5, 20-031 Lublin, Poland; aleksandra.kowalska@umcs.lublin.pl

**Keywords:** fraud, unrecorded alcohol, illicit, alcohol, methanol, policy

## Abstract

Illicit (unrecorded) alcohol is a critical global public health issue because it is produced without regulatory and market oversight with increased risk of safety, quality and adulteration issues. Undertaking iterative research to draw together academic, contemporary and historic evidence, this paper reviews one specific toxicological issue, methanol, in order to identify the policy mitigation strategies of interest. A typology of illicit alcohol products, including legal products, illegal products and surrogate products, is created. A policy landscape matrix is produced that synthesizes the drivers of illicit alcohol production, distribution, sale and consumption, policy measures and activity related signals in order to inform policy development. The matrix illustrates the interaction between capabilities, motivations and opportunities and factors such as access, culture, community norms and behavior, economic drivers and knowledge and information and gives insight into mitigation strategies against illicit alcohol sale and consumption, which may prove of value for policymakers in various parts of the world.

## 1. Introduction

Alcohol is consumed before, during and after meals; to celebrate birth and mourn death; to socialize and is used as a relaxant and as a deliriant [1]. Globally, alcohol use is one of the important risk factors for non-communicable human disease [2,3]. The harmful use of alcohol results in around 2.5 million deaths a year, and alcohol consumption is the third-highest risk factor for disease and disability. In middle-income countries, alcohol is the biggest risk factor, often related to multiple social problems, including dependence [4]. A reduction in alcohol consumption is associated with a lower risk of heart disease and cancer. There are some studies that indicate that moderate alcohol intake has a preventive effect on cardiovascular disease [5], but negative consequences of regular consumption of alcohol often exceed the benefits. Alcohol is addictive, lacks nutrition value and may be a key cultural component in the human obesity dilemma [6,7], but the exact impact is unclear [8,9].

Global consumption of alcohol in 2005 was an average of 6.13 L of pure alcohol per individual aged 15 years or over, with 28.6% of this amount being unrecorded alcohol, i.e., illegally produced or homemade or sold outside government controls and jurisdiction [4]. The World Health Organization (WHO) estimates consumption increased to 6.4 L in 2016, an increase of 4.4% compared to 2005 [10]. Drinking patterns and associated social norms vary between countries and social groups, and, consequently, the harmful use of alcohol disproportionately affects certain individuals, families and communities more than others [11]. In 2015, European regions had the highest prevalence of heavy episodic alcohol use [2], six times more per capita than Southeast Asia and 20 times more than the Eastern Mediterranean, the region with a high Muslim majority [4]. Cultural norms of abstinence in some communities, often driven by religious beliefs and restrictions, influence the social norms around alcohol consumption [12]. When considering deaths attributed to alcohol, the more significant health burden is with men, showing 7.4% of all male deaths being attributed to alcohol consumption compared to 1.4% with women, and lower socioeconomic status and educational levels are linked to a greater risk [4,13].

Over a quarter of the total alcohol consumed globally is unrecorded, illicit or otherwise described as unreported [3,10]. The WHO describes unrecorded alcohol as “alcohol that is not taxed and is outside the usual system of governmental control, because it is produced, distributed and sold outside formal channels” [5].

Whilst the proportion of unrecorded alcohol in Europe is about 21.9% of total per capita alcohol consumption, this rises to 56.2% in the Eastern Mediterranean and to 69% of consumption in Southeast Asia [4,14]. The proportion of unrecorded alcohol as a percentage of total alcohol is as high as 59% in Bhutan, 44.4% in Kuwait, 42.3% in Uganda and 40.1% in the Republic of Moldova [3]. Thus, illicit alcohol sales form a large proportion of total sales in many countries, are unregulated and there is an associated public health risk that is worthy of further research. Undertaking an iterative narrative review of the literature to draw together academic, contemporary and historic evidence, this paper reviews one specific toxicological issue associated with unreported alcohol, methanol contamination, in order to identify the policy mechanisms of interest that can be explored in further research. A typology of illicit (unrecorded, unreported) alcohol products is created, and a policy landscape matrix synthesizes the drivers of illicit (unrecorded, unreported) alcohol production, distribution, sale and consumption in order to inform policy development.

## 2. Illicit Alcohol Production

Alcohol is one of the top four most reported fraudulent commodities after meat, seafood and milk [15]. Illicit alcoholic products are a significant health challenge, especially where adulterants, such as methanol, have the potential to cause harm [16,17]. Adulteration is described here as when a drink contains additional material, such as methanol, or is adjusted using extraneous, substandard or inferior ingredients which are often undeclared to the purchaser, thus rendering the product fraudulent [18,19]. Activities to circumvent religious restrictions, alcohol-related taxes or simply individuals motivated by an economic gain to produce and then sell illicit products have led to the multiple fatal case study incidents that form the focus of this study. False declaration associated with wine is a major issue of concern [20,21,22]. Adulteration of alcohol includes the non-disclosed use of ethylene glycol and methanol to fortify (raise the alcohol level) and/ or improve taste [23,24,25,26]. However, despite improved detection methods [27,28,29], the adulteration of alcohol products remains a concern at local, national and international scales. The 2018 European Union (EU) Report on the EU customs enforcement of intellectual property rights (IPR) shows that there has been a significant increase (>50% increase compared to 2016) in the numbers of alcohol beverages detained at the EU border in 2017 [30]. The Republic of Moldova was the main country of provenance for alcoholic beverages suspected of infringing one or more intellectual property rights (IPR) arriving in the EU.

Traditionally, discussion on the food safety issues associated with alcoholic beverages has focused on chemical and physical food safety hazards such as glass or metal from the processing line. Carcinogenic components in alcoholic beverages, such as acetaldehyde, ethyl carbamate, formaldehyde and acrylamide are of concern as well as heavy metals being present [31,32]. However, one of the main concerns is methanol, and this is now considered.

Homemade or informally produced alcoholic beverages are mostly fermented beverages made from sorghum, millet, maize, rice, wheat or fruits [4]. Methanol can be produced in the fermentation process, and its presence along with ethanol in distilled spirits might be a health hazard [26]. The consumption of methanol causes not only death, but also blindness [24], although the problems usually stem from illegal methanol addition to spirits (alcoholic drinks). Methanol is also a raw material found in a variety of products, including antifreeze, solvents, paints, varnishes [25,26], hand sanitizer, cough mixtures, rubbing products and mouthwash, so if these products are intentionally consumed, it can prove fatal. Antifreeze, windscreen wash fluid and other products containing ethylene glycol and methanol are low-cost [24] compared to alcohol and freely available globally. Direct consumption of alcohol-based products has also led to fatalities, including consumption of cologne/perfume [33,34], bath lotion [35], methylated spirits [36] and direct consumption of windscreen wash fluid [37]. In their research from 1992 to 2001 in Turkey, Yayci et al. [33] note a gender influence with methanol poisoning, with men having the predominant fatalities (89.1%) compared to females (10.9%). In Africa, methanol poisoning from illicit alcohol production and sale is a particular health concern [23,38,39,40]. In 1963 in Spain, methanol was used to adulterate mixed alcohol liqueurs, and this incident led to 51 deaths [41]. However, this issue is also a contemporary challenge, as in Iran in 2018, 76 people died, 460 were hospitalized and 768 were made ill from a methanol-poisoning incident [42]. These illicit alcohol products are made in a domestic setting or in semi-industrialized illegal stills [23], and during the COVID-19 pandemic, some false and misleading information about the positive effect of drinking alcohol on preventing or curing a possible infection was disseminated in (social) media [43]. This resulted in a methanol-related mass poisoning outbreak in Iran, where nearly 300 people died in March 2020 [44]. For a wider perspective of the impact of methanol poisoning, a search of academic and grey literature to determine public health incidents (n=68) associated with methanol-related poisoning is synthesized in Table 1. This table has been collated before the COVID-19 outbreak so that the potential impact of the pandemic on the supply chain and social behavior is excluded from the analysis.

This historic and contemporary evidence positions the social and economic impact of illicit alcohol supply where methanol is the key adulterant. The next section of the paper considers the methodology used and how to create a typology for illicit alcohol products.

## 3. Materials and Methods

The aim of this iterative narrative review is to critique existing literature and frame the context of illicit (unrecorded) alcohol production that is emergent from the academic and grey literature searches. A case-study-based narrative is developed to identify the nature of the incident, country, year incident occurred and the number of casualties (Table 1). The cases are designed to be qualitative and indicative rather than a quantitative representation. Search terms such as “alcohol AND illegal AND unreported AND methanol AND deaths AND casualties” were used to create a snowball academic literature review until data saturation was reached, i.e., no more incidents could be found or further material did not add to the emergent narrative or evidence base. The search was undertaken in the English language only. This is a limitation of the study because, in many countries where methanol poisoning is a public health issue, English is not the first language. However, the common language of the researchers was English. Further work could be undertaken in the future, extending the search string and then searching in a range of languages. The databases used in the search were Science Direct, Google Scholar and Google for the grey literature sources. There was no limitation on age of source in the search, but relevance was considered and any sources deemed not to be relevant were excluded. The case study approach allows for a more holistic inquiry that seeks to be exploratory, explanatory and descriptive [57] in order to drive a causal investigation [58]. Case study analysis is an accepted method for considering business fraud [59,60,61]. A limitation of this approach is the risk of selection bias, and this is considered in the analysis of the findings.

The second stage was to develop a typology of illicit alcohol products. The typology uses four categories. Illicit alcohol products can be summarized into four main categories: (1) illegally produced or smuggled alcohol products (including illegal homemade alcohol), (2) alcohol products that are legitimate, but not in the jurisdiction of their consumption, (3) legal but homemade and (4) surrogate non-beverage alcohol products not intended for human consumption, e.g., industrial alcohol or alcohol-based mouthwash, perfume, etc. [62,63]. These characterizations focus on the products themselves rather than considering their modes of distribution. Illicit trade can also be considered in terms of both the product (legal/illegal) and the modes of distribution (legal/illegal; within-borders or cross-borders), i.e., (1) legal products being illegally distributed within national boundaries, (2) illegal products being distributed within national boundaries, (3) legal products being illegally distributed across borders and (4) illegal products being distributed across borders [64]. Based on these elements and the incidents in Table 1, a typology has been developed (Table 2) that extends product type and product description and considers mode of distribution either within national borders or between countries.

## 4. Results

Within the typology, there are three categories where illicit alcohol is produced, distributed, sold and consumed: legally produced products that can then access an alternative or illicit market/supply chain; illegally produced products that can be sold in an alternative supply chain or can pass into a legal supply chain and third, surrogate products that are not produced for human consumption. It is worth noting that the production of illicit alcohol is often carried out in unhygienic and uncontrolled conditions, and contraband/smuggled alcohol products are beyond the safeguards of the official control of imported foodstuffs. Workers in facilities producing illicit alcohol, and the general public in the area, can be exposed to the risk of industrial accidents, e.g., explosion [65]. Therefore, having described the typology, how can governance frameworks be developed to address illegal alcohol production and sales?

**Figure 1:**
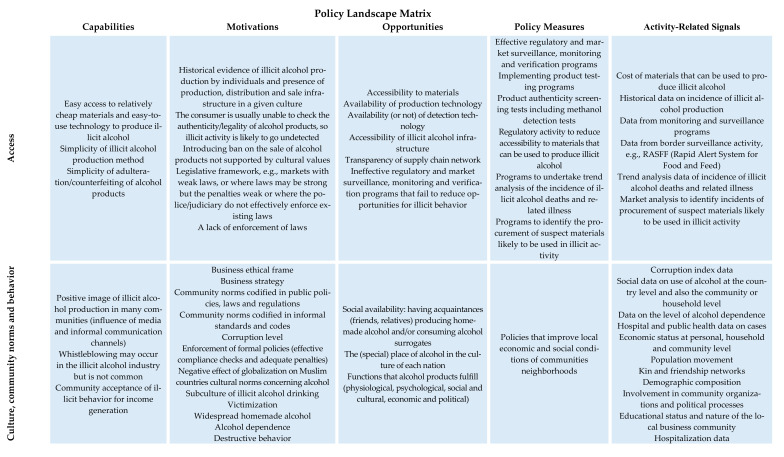

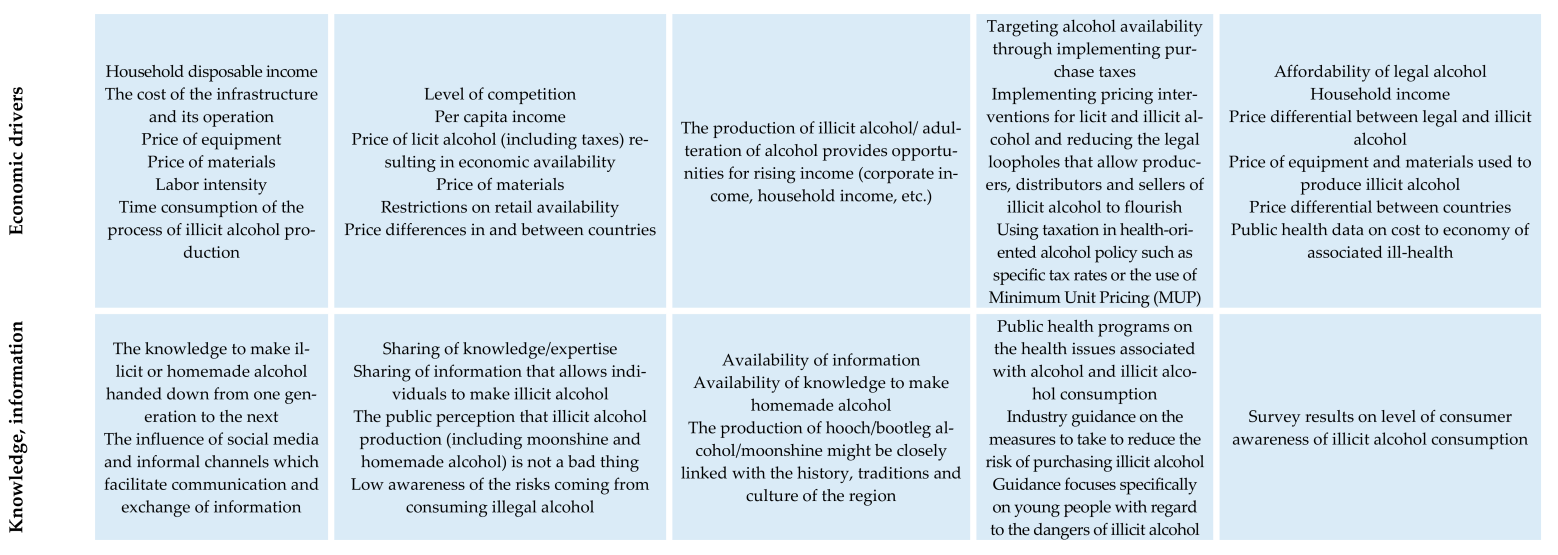
Drivers of illicit alcohol production, distribution, sale and consumption, policy measures and activity-related signals (Adapted from [38,65,69,70,71,72,73,74,75]).

There is a strong economic driver for individuals and organizations to engage in illicit practices [16]. Factors that frame and incentivize this activity include weak public and private institutions, corruption, low Gross Domestic Product (GDP), low tax morale, high taxes or complex tax systems [66,67] and the price differential between illegal and legal alternatives [67]. The classic “fraud diamond” model proposes that four factors influence the potential for illicit behavior: motivation, capability, opportunity and pressure [68]. The main **motivation** for illicit behavior may be the economic gain derived, to circumvent cultural or religious restrictions on access to alcohol and/or to support an individual’s own alcohol dependence. **Capability**, i.e., the ability of an individual or organization to undertake deceptive activities requires both the knowledge and equipment to produce alcohol for home use, evading the associated taxes and excise duty or otherwise to distribute and sell illicit alcohol. The **opportunity** to supply illicit alcohol, either to themselves or others, is also a factor of influence and such opportunity is mediated by the level of regulatory governance in particular countries. Thus, there are both economic and social drivers of illicit alcohol production, distribution and sale, and these form **pressure** that leads to the development of socioeconomic networks with inter-related strategies, activities and dynamic components that drive illicit alcohol consumption or other forms of alcohol-based product abuse [69]. In order to understand these drivers and their interrelationship in more depth, a conceptual policy landscape matrix has been postulated (Figure 1) that illustrates the interaction between capabilities, motivations and opportunities and factors such as access, culture, community norms and behavior, economic drivers and knowledge and information. Pressure was not taken into consideration as a single issue here, but seen to be embedded implicitly in all aspects of the policy landscape matrix. The matrix provides an opportunity to consider policy implications for reducing illicit alcohol production, distribution, sale and consumption, policy measures that could be employed at the state (public) and the market (private) level and the potential data sources (signals) that can arise.

The policy implications are addressed in the four areas of the policy landscape matrix (Figure 1): with policy measures related to access, knowledge and information; economic drivers and culture; community norms and behavior—and these are now considered in turn.

**Access-related policy measures**

Effective regulatory and market surveillance, monitoring and verification programs reduce opportunities for illicit behavior to remain undetected. Policy measures such as implementing product testing programs as part of a wider policy initiative will identify harmful alcohol at the point of production, sale or distribution. Non-targeted product authenticity screening tests are of value as well as targeted adulterant-specific testing such as for the presence of methanol [76,77,78]. A coordinated surveillance program is required across specific trading areas such as the EU; otherwise, if one member state is contributing less to systems such as the RASFF database, or there is a variance in national arrangements of food control systems (in accordance with “Official Control Regulation”), this creates the possibility for a member state to become a “back door” for allowing illicit alcohol products to then have free movement of food within the EU [79,80]. Regulatory activity to reduce accessibility to materials that can be used to produce illicit alcohol should be introduced as well as programs to identify the procurement of suspect materials likely to be used in illicit activity. Further, as part of wider public health surveillance, there should be programs adopted to ensure trend analysis of the incidence of illicit alcohol deaths and related illnesses through the integration of public health data from hospitals and the community.

**Knowledge- and information-related policy measures**

Following the methanol poisoning in Iran in 2018, Aghababaeian et al. [42] state that incidents often occur in low-income Islamic countries, and so effective educational programs are required to raise public awareness of the health issues involved. These programs can use a range of media and communication channels to explain the dangers of production, consumption, distribution and sale of illicit alcohol. Abramowicz et al. [80] underline in their study that activities undertaken via education/prevention schemes should be aimed at a particular group of consumers, appropriately profiled and fully tailored to their needs. Growing children and adolescents are a key target group here since they often undertake new forms of behavior and experimentation, including using alcohol, in order to determine their place in society [81].

Industry guidance on the measures to take to reduce the risk of purchasing illicit alcohol is also important. Shapira et al. [82], in their study on methanol levels in illegal alcoholic beverages sold in a low socioeconomic area of Tel-Aviv, state there is a need to inform shopkeepers about labeling regulations and “make information and health warnings accessible to the foreign-born population residing in the area,”, i.e., that information must be accessible, context-specific and available if required in a range of languages. The more alcohol marketing that young people are exposed to, the more alcohol they will consume; indeed, restrictions on access to alcohol for young people may actually promote this illicit parallel market [83], creating an “underground economy” as demand remains the same, but can no longer be met through legal supply routes.

**Economic-related policy measures**

Economic related policy measures that have, or could be adopted include: targeting alcohol availability, implementing purchase taxes [38], implementing pricing interventions for licit and illicit alcohol and reducing the legal loopholes that allow producers, distributors and sellers of illicit alcohol to flourish [70]. In Europe, there is a long-standing debate about the effectiveness of using taxation in health-oriented alcohol policy such as specific tax rates or the use of Minimum Unit Pricing (MUP) [75], as in Australia and the UK, including Scotland [84]. In Canada, Social Reference Prices (SRPs) for alcoholic beverages, i.e., “floor” or “minimum” prices for a given “unit” or “standard drink”, have been set [85]. As inter alia alcohol tax regimes vary across territorial domains and product categories; this leads to differential pricing of similar products between markets and between products categories within a given market. This might increase the incentive for illicit behavior.

The main opposition to this policy approach comes from the alcohol industry and free-market-oriented think tanks [84]. Transnational alcohol corporations, in contrast to tobacco corporations in their market sector, continue to have a significant impact on alcohol policy globally [75,86,87]. As a rule, consumption of licit alcohol declines as price increases [71]. Consumption of illicit alcohol may grow as licit alcohol price increases; however, raising taxes on licit (recorded) alcohol together with reinforcing measures against unrecorded alcohol might lead to a decline in total alcohol consumption. Moreover, when restrictions are placed on the retail availability of licit alcohol, whilst consumption of licit alcohol decrease [71], consumption of illicit alcohol may increase unless the protection against unrecorded alcohol sale and consumption is strengthened. Furthermore, producers of illicit alcohol tend to increase prices when recorded alcohol beverages prices are on the rise. These phenomena limit the effectiveness of economic policy measures and make it hard to find the optimum fiscal solution.

**Culture, community norms and behavior-related policy measures**

Illicit alcohol use, as with drug use, is associated with specific social networks, so social causation and neighborhood mitigation processes may discourage illicit alcohol use [72]. Madureira-Lima and Galea [88] created an Alcohol Control Policy Index (ACPI), including policies from the WHO’s Global Strategy to Reduce the Harmful Use of Alcohol. These are leadership, awareness and commitment; health services’ response; community action; drink driving policies and countermeasures; availability of alcohol; marketing of alcoholic beverages; pricing policies; monitoring and surveillance, reducing the negative consequences of drinking and alcohol intoxication and the focus of this study, reducing the public health impact of illicit alcohol and illegally produced alcohol. In Iran, 9 out of the 10 policy measures have been introduced, excluding pricing policy [89]. However, some communities, especially low-income groups, have an innate cultural relationship with illicit alcohol consumption, and in these social groups, addressing illicit alcohol within a wider alcohol management plan is of value [90,91]. Thus, for policies to be effective, they need to reflect the social context in which they are adopted.

**Early warning systems (EWSs) to reduce illicit alcohol sale and consumption**

The development of an EWS is an essential policy measure to mitigate illicit alcohol sale and consumption. EWSs allow health officials to be alerted so they can minimize the health impact of an illicit alcohol or methanol incident on the population. Recognizing the types of signals of concern underpins the development of an EWS for illicit alcohol sale and consumption. Some signals may be weak, i.e., imprecise early indicators of an impending event, or they may provide stronger evidence of a potential incident [92,93]. The process of developing an EWS can be broken down into the following elements:

**Monitoring phase**—considering specific criteria and ensuring the data can be collected and is of the required granularity;
**Analysis phase**—assessing data, indicators, trends in order to be able to differentiate critical events;
**Prediction phase**—depending on the level of criticality, early warning information is generated and communicated to relevant stakeholders; and
**Implementation phase**—appropriate measures are defined and implemented [94].

Signal detection theory reflects the challenging issue of detecting a given signal against a background of noise, i.e., in a situation of uncertainty [95]. Signals or indicators can be monitored to determine any trends and if these trends give cause for concern (Phase 1). The analysis phase (Phase 2) requires signals to be assessed to determine any associations with other variables, e.g., gender, age, location, frequency, distribution, symptoms, duration of illness, severity and outcome [96]. The policy landscape matrix developed in this research requires the translation of discrete signals and their amplification to develop a risk signal. The signal can be characterized by its degree of relevance and also by its strength, i.e., the magnitude of evidence, single or multi-dimensional [97]. The signal. as a result, can be validated to ensure that the information received is sufficient to suggest causal association and support further action based on the information. Thus, regulatory sampling can provide some signals, but other signals will come from both social and economic factors that influence illicit behavior. Signal detection and the wider policy program need to be linked to sufficient resources that underpin information systems, policy measures and reporting systems designed to reduce the risk of illicit alcohol to public health.

In 2018, the WHO launched the SAFER initiative alongside the United Nations’ third high-level meeting on prevention and control of non-communicable diseases (NCDs) to provide support in reducing the harmful use of alcohol through: (1) strengthening restrictions on alcohol availability; (2) advancing and enforcing drink-driving countermeasures; (3) facilitating access to screening, brief interventions and treatment; (4) enforcing bans or comprehensive restrictions on alcohol advertising, sponsorship and promotion; (5) raising prices on alcohol through excise taxes and other pricing policies [98]. The WHO’s document suggests eradicating illicit alcohol or bringing it under government control in countries where informal markets are the main source of alcohol. Another suggestion is to develop tax policies that make low-alcohol and non-alcoholic variations of culturally preferred beverages more attractive and to introduce tax stamps to show that duty has been paid on informal products.

## 5. Conclusions

Illegal and unrecorded alcohol and its illicit substitutes lack the regulatory and market oversight that legal alcohol products would have, increasing the risk of safety, quality and fraud issues. As illicit alcohol is produced without the management controls and verification systems that are used in the legitimate supply chain, it is a cause of global concern as it presents a clear personal risk to those that consume it. This research has drawn together academic, contemporary and historic evidence on the impact of illicit alcohol production, distribution and consumption. The policy mechanisms that can be explored in further research are identified. A typology of illicit alcohol products is created, and a policy landscape matrix synthesizes the drivers of illicit alcohol production, distribution, sale and consumption in order to inform policy development. Policy measures are addressed in four areas: (1) access; (2) culture, community norms and behavior; (3) economic drivers; and (4) knowledge and information. Methanol, one of the main agents that cause alcohol-related disability or fatality, is shown in this work to be a significant and widely distributed concern as a food-related toxin with global impact. This public health harm needs to be addressed by concerted action at regulatory and market levels. Further, the level of reported illicit alcohol-related health incidents identified in the academic literature, grey literature and media sources described herein has provided strong supporting evidence within a synthesized timeline of the locations and size of this global public health problem.

## Figures and Tables

**Table 1:** Examples of toxic methanol incidents (1963–2020) highlighted in the academic literature and the media search (n=68).

Country	Year	Incident	Casualties	Source
Spain	1963	Methanol used in mixed alcohol liqueurs	51 died	[41]
Papua New Guinea	1978	Mixture of methanol and isopropanol	369 ill; 4 irreversibly blinded; 18 died	[26,36]
Italy	1986	Methanol adulterated wine	90 hospitalized; 23 died	[45]
Cambodia	1998	Methanol poisoning	>400 ill; 60 dead	[46]
China	1998	Methanol poisoning	>200 ill; 27 died	[28,34]
India	1998	Methanol poisoning	97 cases; 28 died	[26]
Madagascar	1998	Methanol poisoning	200 died	[47]
Serbia	1998	Methanol poisoning	>90 ill; 43 died	[28,34]
Bangladesh	1999	Methanol poisoning	121 died	[34,46]
Kenya	1999	Methanol poisoning	24 died	[34,46]
Bangladesh	2000	Methanol poisoning	>100 ill; 56 died	[28,46]
Canada	2000	Methanol poisoning	>12 ill; 2 died	[34]
El Salvador	2000	Methanol poisoning from low-quality alcohol	>200 ill; 117 died; 19 ill; 19 died	[28,34,46]
Estonia (Pärnu)	2001	Illegal spirits with 50%–100%	154 ill; 68 died	[26,28,34,48]
India	2001	Methanol poisoning	>120 ill; 27 died	[28,34]
Kenya	2001	Methanol poisoning	120 died	[34,46]
Madagascar	2002	Methanol poisoning	40 ill; 11 died	[28,46,47]
Norway	2002–2004	Methanol poisoning	59 ill; 17 died	[28]
Saudi Arabia	2002	Methanol poisoning	19 died	[34,46]
Taiwan	2002	Methanol poisoning	9 died	[34]
Botswana	2003	Methanol poisoning	>45 ill; 9 died	[28,34]
Tunisia	2003	Methanol poisoning	16 ill; 3 died	[26]
Iran	2004	Methanol poisoning	62 ill; 17 died	[28,34]
Kenya	2004	Methanol poisoning	23 died	[34]
Turkey	2004	Methanol poisoning	21 died	[34]
Kenya	2005	Methanol poisoning	174 ill; 49 died	[28,34]
Russia	2005	Methanol poisoning	33 died	[34]
Turkey	2005	Methanol poisoning	23 died	[34]
Iran	2006	Methanol poisoning	42 ill; 6 died	[34]
Nicaragua	2006	Methanol poisoning	801 ill; 48 died	[28,34]
Russia	2006	Methanol poisoning (2 incidents)	60 ill; 3 died; 13 died	[28,34]
India	2008	Methanol poisoning	285 ill; 150 died	[28,34]
Mongolia	2008	Methanol poisoning	>32 ill >11 died	[34]
India	2009	Methanol poisoning	63 ill; 20 died >275 ill 136 died	[28,34]
Indonesia	2009	Methanol poisoning	45 cases; 25 died	[28,34]
Uganda	2009	Methanol poisoning	77 ill; 27 died; 189 ill; 89 died	[28,34]
Cambodia	2010	Methanol poisoning	17 died	[34]
India	2010	Methanol poisoning	10 died	[34]
Indonesia	2010	Methanol poisoning	5 ill; 3 died	[34]
Kenya	2010	Methanol poisoning	>17 died	[34]
Uganda	2010	Methanol poisoning	189 ill; 89 died	[34]
Ecuador	2011	Methanol poisoning	>770 ill; 51 died	[28,34]
Haiti	2011	Methanol poisoning	40 ill; 18 died	[28,34]
India	2011	Methanol poisoning (multiple incidents)	>370 ill; 170 died; >167 ill; 143 died; 100 ill; 31 died	[28,34]
Kenya	2011	Methanol poisoning	29 died	[34]
Russia	2011	Methanol poisoning	19 ill; 4 died	[34]
Sudan	2011	Methanol poisoning	>137 ill; 71 died	[28,49]
Turkey	2011	Methanol poisoning	22 ill; 5 died	[34]
Cambodia	2012	Methanol poisoning from contamination of rice wine	367 ill; 300 hospitalized; 49 people died	[28,34]
Czech Republic	2012	Methanol poisoning	121 hospitalized; 41 deaths	[50,51]
Honduras	2012	Methanol poisoning	48 ill; 24 died	[28,34]
India	2012	Methanol poisoning	37 ill; 17 died; 100 ill; 31 died	[34,52]
Iran	2013	Methanol poisoning	694 ill; 8 died	[28]
Libya	2013	Methanol poisoning from illegal alcohol	1066 ill; 101 deaths	[25]
Pakistan	2013	Methanol poisoning from illegal alcohol	8 deaths	[25]
Kenya	2014	Two incidents of methanol poisoning	Incident 1—341 ill; 100 dead; Incident 2—126 ill; 26 dead	[25]
Nigeria	2015	Methanol poisoning from a local beverage	89 dead	[51]
Turkey	2015	Methanol poisoning	32 dead	[26]
Russia	2016	Methanol poisoning from consumption of bath lotion	57 hospitalized; 49 died	[35]
Iran	2018	Methanol poisoning 7 September–7 October 2018	768 ill; 460 hospitalized; 76 died	[42]
Malaysia	2018	Methanol poisoning from counterfeit alcohol	45 died	[53]
India	2019	Methanol poisoning	130 died	[53]
Costa Rica	2019	Methanol poisoning	20 died; 45 ill	[54]
Dominican Republic	2019	Methanol poisoning (10 tourists in 12 months)	Around 10 deaths	[55]
Malaysia	2019	Methanol poisoning (3 clusters)	6 died; 19 ill	[56]
Iran	2020	Methanol poisoning as the result of COVID-19 outbreak	296 died, 2197 ill; 824 hospitalized	[44]

**Table 2:** Typology of illicit (unrecorded, unreported) alcohol production (Adapted from [62,63,64]).

Product Type	Legal Products		Illegal Products		Surrogate Products	
Product examples	Homemade or informally produced alcoholic beverages or Product smuggled from country where product was legal to an alternative market		Counterfeit or informally produced product		Antifreeze, bath lotion, cologne, methylated spirits, mouthwash, windscreen wash	
Product description	Homemade and legal for home consumption but not for sale	Legal in country of production but not in country of consumption	Illegally produced in country of consumption at home or larger scale manufacturing	Illegally produced in country of production and transferred illegally (smuggled) to country of consumption	Legally produced in country of consumption but not for human consumption	Legally produced in country of consumption but not for human consumption and then exported
Distribution	Within national boundaries	Across borders	Within national boundaries	Across borders	Within national boundaries	Across borders

## Data Availability

Data sharing not applicable.
